# Sodium Reduction in Bouillon: Targeting a Food Staple to Reduce Hypertension in Sub-saharan Africa

**DOI:** 10.3389/fnut.2022.746018

**Published:** 2022-02-02

**Authors:** Nicholas S. Archer, Maeva Cochet-Broch, Mihaela Mihnea, Gonzalo Garrido-Bañuelos, Patricia Lopez-Sanchez, Leif Lundin, Damian Frank

**Affiliations:** ^1^Commonwealth Scientific and Industrial Research Organisation (CSIRO) Agriculture and Food, Sydney, NSW, Australia; ^2^RISE Research Institutes of Sweden, Agriculture and Food, Gothenburg, Sweden; ^3^Commonwealth Scientific and Industrial Research Organisation (CSIRO) Agriculture and Food, Melbourne, VIC, Australia

**Keywords:** reformulation, flavor perception, chloride salts, flavor enhancement, cross-modal interaction, fortification

## Abstract

Bouillon cubes are a staple ingredient used in Sub-saharan African countries providing flavor enhancement to savory foods. Bouillon has been identified as a vehicle for fortification to overcome micronutrient deficiencies in Sub-saharan Africa. However, bouillon has a high sodium content (and in addition with other foods) contributes to dietary sodium intake above recommended guidelines. High dietary sodium intake is a key risk factor for hypertension and cardiovascular disease (CVD). Africa has the highest rates of hypertension and CVD globally with nearly half the adult population above 25 years affected. This review presents current state of research on sodium reduction strategies in bouillon. The key challenge is to reduce sodium levels while maintaining optimal flavor at the lowest possible production cost to ensure bouillon continues to be affordable in Sub-saharan Africa. To produce lower sodium bouillon with acceptable flavor at low cost will likely involve multiple sodium reduction strategies; direct reduction in sodium, sodium replacement and saltiness boosting flavor technologies. Efforts to reduce the sodium content of bouillon in Sub-saharan Africa is a worthwhile strategy to: (i) lower the overall sodium consumption across the population, and (ii) deliver population-wide health benefits in a region with high rates of hypertension and CVD.

## Introduction

The culinary concept of bouillon is to create an intense flavourful extract or essence from cooked meat and animal products (and/or vegetables). The sodium content of bouillon is high (mainly from salt) and provides a significant contribution to the flavor enhancement of bouillon. Sub-saharan Africa has broad use and frequent consumption of bouillon. The objective of this review is to examine the evidence for the contribution of bouillon to total dietary sodium intake in Sub-saharan Africa, the current regulation/initiatives for sodium levels in bouillon and review strategies for sodium reduction through bouillon reformulation as a means to contribute to improved population health in Sub-saharan Africa.

## Dietary Sodium Intake and Bouillon in Sub-saharan Africa

### African Dietary Sodium Intake and Health Impact

Sodium is an essential nutrient necessary for normal body function. The minimum sodium intake required for an average adult is ~0.5 g per day. However, the level of dietary sodium intake worldwide is above the World Health Organization (WHO) recommended amount of 5 g of salt (sodium chloride, NaCl) a day (~2 g sodium a day) (1, 2). Long term intake above these levels is associated with negative health consequences. In 2010, the estimated global mean salt intake was 10.06 g/day (1). While African regions in general are reported to have lower sodium intake compared to other world regions, the average amount consumed across all African regions is above the WHO recommended intake guidelines (1, 3). Where studies have assessed intake in specific African countries, the reported salt intake is more comparable or above to the mean global intake level of 9–10 g/day (4–9). Furthermore, sodium intake is likely to rise in African populations in the future (similar to other developing countries) as dietary patterns transition to a higher proportion of processed and convenience foods (10, 11). There is limited reliable data regarding the total sodium intake in Sub-saharan Africa and there is a requirement for further country-level research to better understand dietary salt intake levels.

Long term excess sodium intake has serious health consequences. High sodium levels are a key risk factor for increased blood pressure (due to increased water retention) resulting in hypertension and the potential to develop cardiovascular disease (CVD), increased risk of a stroke and other health complications (12). These lifestyle preventable diseases (hypertension, CVD, stroke) are major contributors to the overall prevalence of non-communicable diseases (NCD) which account for the greatest proportion of global morbidity and mortality rates (13, 14). Furthermore, a moderate reduction in sodium intake is associated with lowering blood pressure, and lowering the rate of CVD (15), showing sodium dietary reduction is a key intervention to combat CVD and associated disease burden.

NCD have a higher frequency in low to middle income countries (LMIC). Within Africa, 20 million people suffer from CVD and hypertension affects nearly half the population of adults >25 years, representing the highest incidence worldwide (16–19). Further compounding the hypertension prevalence is the high frequency of individuals who are unaware of their hypertensive status and the low treatment rate for hypertension (20). Both factors work together to cause greater CVD disease severity and secondary complications (e.g., kidney disease) resulting in greater burden on healthcare systems (21). Therefore, salt reduction initiatives in Africa could play a significant role in the prevention and control of high blood pressure and CVD and lower morbidity/mortality rates associated with NCD.

In 2015, WHO released the Sustainable Development Goals (SDGs) outlining 17 global goals to achieve a better and more sustainable future for all (22). Goal number 2: Zero hunger aims to “end all forms of malnutrition, including achieving, by 2025, the internationally agreed targets on stunting and wasting in children under 5 years of age, and address the nutritional needs of adolescent girls, pregnant and lactating women and older persons.” Bouillon cubes are consumed on a daily basis in Central and Western Africa and are an excellent candidate for Large Scale Food Fortification and private public partnership where the food industry take a lead and is supported by the public sector to generate health impact at scale (23, 24).

In addition, a reduction in dietary sodium intake is a central component to achieve SDG Target 3.4 which aims is to reduce premature deaths from NCDs by one third by 2030. Specifically, WHO suggests multiple strategies for salt reduction having the greatest cost effectiveness in LMIC including reducing salt intake through the reformulation of food products and, reducing salt intake through a behavior change (25, 26). Furthermore, the WHO Global action plan for the prevention and control of NCDs 2013–2020 has set a target for a 30% relative reduction in mean population intake of salt/sodium by 2025 (Target 4) (27).

Few studies have assessed the health and economic costs of sodium reduction in Sub-saharan Africa. The limited studies completed to date show lowering sodium across the population results in significant direct and indirect health cost savings and health gains (28). Modeling in South Africa show sodium reduction (through implementation of mandatory salt targets) would result in 11% reduction in CVD deaths and lower overall government health costs by 0.32% (29). Likewise a recent study in Cameroon show a 30% reduction in sodium intake is associated with 16.8% reduction in premature CVD mortality and 776,400 health-adjusted life years from 2016 to 2030 (30). The direct and indirect economic costs of hypertension in Sub-saharan Africa requires further studies to estimate economic costs. Findings from systematic reviews and meta-analyses highlight the requirement for more high-quality studies to show the impact of different salt reduction initiatives (i.e., product reformulation or consumer education) on reducing the incidence of hypertension and CVD in Africa (31, 32).

#### Source of Dietary Sodium Intake in Sub-saharan Africa

There are three main sources of dietary sodium intake: (i) naturally occurring sodium present in non-processed foods, (ii) added sodium in packaged or processed foods, and (iii) sodium added during cooking or during eating at the table. The sodium reduction strategies employed on a country/regional basis should target the largest source of discretionary salt intake.

In developed countries, the majority of sodium is consumed from processed foods (75–80%) with small contributions from sodium found naturally in foods or sodium salt added during and at the end of cooking (33). Therefore, sodium reduction measures in developed countries have focused on reducing sodium in ready to eat and packaged foods (34). These measures have already, and will continue to, have great success to reduce sodium intake from commonly consumed foods, particularly where governments have set legislative targets for sodium reduction.

Similar strategies to reduce sodium intake that have been successful in developed countries will not likely translate to other LMIC nations, as the contributors to sodium intake differ significantly. In Sub-saharan African countries, seasoning of foods during cooking is reported to be the major contributor to sodium intake, for example use of salt, soup mix or bouillon cube added while cooking (1, 5, 6, 34). Furthermore, several surveys of African countries report a low frequency (~10%) of sodium addition to foods after cooking or at the table (16, 35). Therefore, strategies targeting a reduction in salt during cooking are likely to have the largest impact on lowering sodium intake.

Bouillon cubes are a staple food in Sub-saharan African countries (particularly West and Central Africa) and could be a key target for sodium reduction. Bouillon has a high rate of consumption across country, age, gender and socio-economic levels and also contains a high sodium content (35–37). While bouillon is consumed regularly across the population (e.g., on average 1.7–4.3 g per person a day) (37–40), it is not known the relative contribution bouillon contributes to daily sodium intake in excess of recommended levels in African countries (in comparison with other foods). However, several publications directly link high daily sodium intake levels in African populations with bouillon cube consumption:

- From Melse-Boonstra et al. (5): “We speculate that the practices contributing to the relatively low contribution of discretionary salt *per se* in Benin [but higher total salt intake] is the use of condiments cubes (Maggi cubes; Nestle SA, Vervey, Switzerland, and similar products from other manufacturers). These are used in sauces eaten with staple foods and are rich sources of salt.”
- Adeyemo et al. (41): on interviews and observation of cooking by South Nigerians “…salt was consistently added to food during cooking but rarely at the table; and there were only a few, widely consumed sources of non-salt sodium—bouillon cubes, fermented locust beans (iri), monosodium glutamate seasoning, and dried, salted fish.”
- Leyvraz et al. (35): “…salt rich condiments (e.g., Maggi cubes and food spreads such as Marmite/Vegemite), which are often added in soups and other dishes, were also used frequently in all counties [Benin, Guinea, Kenya, Mozambique, and Seychelles].”
- Mezue (42): “… the source of salt in the Nigerian diet will likely be from salt added at cooking and on the table, seasoning cubes (such as Maggi and Royco), flavorings [especially those which contain monosodium glutamate (MSG) like Aji-no-moto] and fermented locust beans (Iru or Ogiri)….”
- Queiroz et al. (6): “Sodium from salt and stock powder added to culinary preparations was the most reported contributor for the total intake.”
- Menyanu et al. (9): “…the Ghana National Iodine Survey Report 2015 reported frequent and widespread use of bouillon cubes with nearly half (48.8%) of the participants reporting consumption of >5 times a week.”

This provides evidence that bouillon contributes to daily dietary sodium intake in Sub-saharan African countries above recommended levels. Therefore, decreasing the sodium content of bouillon may contribute to a significant reduction in the overall sodium consumed across the population and contribute to population wide health benefits through reduction in the rate of hypertension and death from CVD.

#### Approaches to Reduce Sodium Levels for Improved Population Health

As outlined, sodium reduction strategies should target the largest sources of discretionary salt intake. There are several approaches to reduce dietary sodium intake across a population, including:

(1) The reformulation of foods having high sodium content with frequent consumption patterns to contain lower levels of sodium and/or,
(2) Increased consumer education about common foods high in sodium (including bouillon cubes) and the negative health impacts from a diet high in sodium (therefore driving behavior change to reduce sodium intake).

Reducing sodium intake at a population level to achieve health impacts will require a whole food system approach, for e.g., greater health gains can be achieved if interventions are targeted across the food system, of which bouillon should contribute given the evidence of high consumption patterns. Indeed, there have been several intervention studies aimed at reducing dietary sodium intake (which have included bouillon) showing positive impact on health outcomes (41, 43). In a randomized controlled trial, Charlton and colleagues found significant reduction in systolic blood pressure after an 8-week intervention where participants were provided with five commonly consumed food items with reduced sodium levels (bread, margarine, bouillon, soup mix, and flavor enhancer), compared to control group who were provided with the same normal sodium level foods (43). The results highlight reformulation of commonly consumed foods with lower sodium levels as an effective strategy for improved population health. Likewise, a reduction in sodium intake and blood pressure measures was observed after a 2-week intervention in hypertensive individuals in Nigeria who were counseled on ways to reduce sodium intake, which included cutting salt use by half and eliminating bouillon use (41). This study shows that nutritional education to limit sodium intake is also a feasible option for population wide sodium reduction.

The remainder of this review will focus specifically on strategies for sodium reduction in bouillon (i.e., through bouillon reformulation) as a direct mechanism to contribute to lowering overall population sodium intake. While consumer education to limit bouillon consumption may reduce population level sodium consumption, this approach is likely to be less impactful as consumer education will take longer to achieve public health benefits (i.e., it is a less direct mechanism to lower sodium levels compared to bouillon reformulation) and as outlined above, may reduce the impact of other population health initiatives that aim to fortify bouillon to overcome micronutrient deficiencies in Sub-saharan African countries (24, 38, 39).

### Sodium Levels in Bouillon

Bouillon cubes typically contain a number of ingredients including salt, taste enhancers (e.g., monosodium glutamate, 5′-nucleotides or yeast extract), fat (vegetable or animal), hydrolysed vegetable proteins, starch, herbs, spices and other flavorings (38). Bouillon cubes are used as a seasoning ingredient and generally added to foods during cooking where they enhance the flavor of savory foods like soups, stews and sauces. Additionally, they are also used crumbled over foods as a seasoning prior to cooking (e.g., marinating meats) or crumbled over a meal prior to serving. Bouillon cubes vary in size from 4 g cubes to larger 10 or 11 g tablets. In addition to bouillon cubes, seasoning powders are also growing in popularity in Sub-saharan African countries.

Salt, added in the form of crystalline sodium chloride (NaCl), is generally the main ingredient and contributes most to the sodium content of bouillon (generally between 40 and 60%). Salt is also a convenient bulking agent and natural preservative. However, salt is not the only source of sodium. There are other ingredients that may also contribute to the overall sodium content, including: meat/chicken flavor extracts, hydrolysed vegetable protein (HVP), vegetable flavor extract, yeast extract, monosodium glutamate (MSG), and disodium guanylate and inosinate salts. These ingredients are present in lower quantities and hence contribute a smaller portion of the overall sodium content of bouillon. Thus, lowering the salt (NaCl) content is the most effective reformulation strategy to reduce sodium levels in bouillon. However, as saltiness is such an important and desirable sensory attribute of savory foods, even small reductions in the amount of salt can have large negative consequences on consumer liking. Therefore, understanding how the sensory loss can be compensated through the use of ingredient/technologies is an important area of research to ensure consumer satisfaction.

Understanding the current average sodium levels in bouillon is an important benchmark to assist with developing appropriate targets for sodium reduction and the strategies and technologies employed to meet those targets. Currently, there is no published analysis of sodium content of bouillon products in different world regions, including for Sub-saharan Africa, and only a few country specific reports (44–46). Two separate publications sample sodium levels in commercial bouillon in West African countries with reported ranges of 20,800–26,100 mg sodium/100 g dry bouillon in Senegal (13 bouillon brands assessed by inductively coupled plasma mass spectrometry (ICP-MS) method) (46) and 16,000–30,400 mg sodium/100 g dry bouillon in Togo (21 samples assessed by Mohr method) (45). Further surveys of sodium levels in bouillon are required to get greater understanding for the ability to reduce sodium in bouillon. If translated across the Sub-saharan African region, the high levels of sodium in bouillon observed in Senegal and Togo, in addition to the high frequency of consumption, suggests bouillon reformulation would be an effective strategy to contribute to population wide reduction in dietary sodium intake.

#### National Based Regulations of Bouillon Sodium Levels

National and international based initiatives are important tools to define maximum sodium levels through voluntary or mandatory standards. These initiatives can provide population level controls to reduce sodium intake and improve public health. In Sub-saharan African countries, there are only a few guidelines or legislation which set benchmark levels of sodium in bouillon. South Africa has implemented mandatory legislation for the reduction of sodium levels across a wide range of processed foods including bouillon. The legislation commenced in 2013, providing specific sodium levels for different food groups to be introduced over time. For bouillon/stock cubes, the legislated sodium levels were <18,000 mg/100 g in June 2016 and then a further reduction below 13,000 mg/100 g in June 2019 (47, 48). Senegal and Burkina Faso have set the maximum salt (NaCl) content in bouillon to be no more than 55% of the total bouillon cube (equivalent to 22,000 mg sodium/100 g). Finally, Nigeria has released national food and dietary guidelines to educate the population. The adult guidelines advise to limit the intake of salt, bouillon cubes, and sugar (42), however, there is no maximum salt guidelines specifically around bouillon.

Other worldwide initiatives include: (i) the Pan American Health Organization (PAHO) has set regional levels/guidelines for sodium levels for 11 food categories (Smart Salt Consortium). Regional sodium targets for bouillon are 20,500 mg/100 g and a lower target of 18,000 mg/100 g (49). (ii) Argentina has enacted legislation which sets maximum sodium levels in three processed food groups (came into law in 2014). For bouillon cubes, the maximum sodium content level is 430 mg/100 g (equivalent to ~17,200 mg/100 g dry bouillon) (50). The legislation allows for a further decrease in sodium content over time. In 2019, the bouillon level was revised a further 5% lower, with companies having 18 months to meet revised maximum levels. (iii) Worldwide, there are many other voluntary based initiatives to reduce sodium levels across all foods which include bouillon, for example UK has maximum targets of 15,000 mg/100 g dry bouillon (51).

Where legislated or voluntary sodium reduction levels have been introduced, the reduction has been completed in a stepwise approach of multiple smaller increments over time with significant lead time (2–5 years) provided per reduction step. The differences in legislated sodium levels in African countries of 22% in Senegal and Burkina Faso compared to 13% in South Africa are significantly large and demonstrate a strong role for industry and government leadership in long term sodium reduction strategies. Further, the implementation of low sodium targets on bouillon by South Africa and Argentina shows that it is technically feasible to reduce sodium in bouillon at significantly lower levels than current average sodium levels across Africa. The challenge is to complete this in the most affordable way while maintaining consumer acceptance.

#### Food Industry Sodium Reduction Initiatives

In addition to government legislation, the food industry has also committed to lowering sodium levels in their products. Multinational companies are recognizing sodium levels in the products contribute to excess dietary sodium intake and are showing leadership in product reformulation to reduce sodium. Several multinational food companies dominate the African bouillon market including Maggi (Nestle), Knorr/Royco (Unilever), and Jumbo (GB Foods) (38). Each of these companies have their own initiatives and commitments to reducing sodium levels.

Unilever has developed a salt reduction position and has set benchmarks for sodium levels for different product groups. The benchmarks were formulated/modeled based on a total diet approach considering the contribution of the product group to overall daily salt intake (52, 53). For soups and bouillons, recommended sodium levels of 360 mg/100 g (18,000 mg sodium/100 g dry bouillon cube) is suggested for total dietary intake of 6 g salt/day (2,400 mg sodium) and 265 mg/100 g (13,250 mg sodium/100 g dry bouillon) for 5 g salt/day (2,000 mg sodium) (52).

Nestle has launched the Nestle Nutrition Profiling System (NNPS) developed to guide reformulation of their products (54, 55). The profiling system uses a dichotomous pass/fail approach for each product based on meeting set nutritional target values. Bouillon is categorized into the accessories group and has a target value of ≤ 33% daily sodium intake per serve. The total target value for an adult is 6 g salt/day (2,400 mg sodium). Therefore, bouillon targets are required to be <792 mg/serve ( ≤ 23,760 mg sodium/100 g of dried bouillon cube). The NNPS has been developed based on American/European dietary patterns and has not reported extension of the profiling system in an African context. Translation to African context would require the development of sodium target values for different food groups based on the dietary consumption patterns of the region so the overall amount of salt consumed per day is <6 g/day salt target.

## Functional Aspects of Salt

### Function of Salt in Foods

Sodium salt is a major ingredient used in the food industry and is a core component in all bouillon formulations. The term salt is commonly used to refer to sodium chloride (NaCl) and is composed of 40% sodium and 60% chloride by weight. When crystalline NaCl is dissolved in aqueous solvent (water) it dissociates into free sodium cations (Na^+^) and chloride anions (Cl^−^). Free sodium cations are detected by dedicated taste receptors on the tongue and in other organs and cells throughout the body. The perception of sodium as saltiness is a primary taste modality and is an important and desirable attribute of many foods. Salt is added to foods and beverages (including bouillon cubes) for multiple functions, including:

(1) Taste/flavor enhancement: The addition of salt to food has a significant impact on consumer liking and acceptance. Salt addition to a food results in increased taste/saltiness, boosts/enhances flavor intensity, and can mask other tastes (i.e., bitterness). Although sodium increases the palatability of foods, synergistic interactions of sodium ions with other food components like the amino acid glutamic acid and 5′-nucleotides are well-known to increase saltiness perception and umami or savory characteristics (56, 57).
(2) Preservation/food safety: The growth of pathogens and food spoilage microorganisms can be inhibited by the addition of salt. Salt lowers the availability of water (water activity) which is essential for microbial growth. The addition of salt as a preserving agent is important for both the shelf stability of food products during manufacture, transport and storage/display in a shop and, the safe preparation, cooking, consumption, and storage of a food/meal after the product is purchased by a consumer.
(3) Processability/friability: Salt can provide further product specific attributes. For example, in the production of bread, salt addition assists to control yeast fermentation impacting on bread texture and affects the rate of Maillard reaction flavor formation in baked foods. The presence of salt in powdered food ingredients helps to prevent clumping and increases product flow or friability. In meat products, salt has a significant effect on quality (juiciness, texture, water-holding capacity) as salt solubilises proteins within the myofibrillar network, facilitating water entrapment. In bouillon, salt can contribute up to 50–60% of the product and therefore functions to provide a significant contribution to the size/weight of the cube (in addition to the flavor enhancement). Additionally, bouillon salt is commonly fortified with iodine and has a function to contribute to population wide health to reducing iodine deficiency (39).
(4) Carrier for micronutrient fortification: due to universal consumption across populations, many countries have legislated for salt fortification (table salt and/or in cooking/processed foods). Iodine is the main micronutrient added to salt to prevent iodine deficiency and goiter. In Sub-saharan African countries, the salt in bouillon is commonly fortified with iodine.

Simply reducing the sodium content of processed foods can have far ranging effects not only on the saltiness and flavor, but also other important qualities. Hence, reformulating foods for lower sodium is often a significant and considerable technological challenge. Salt has techno-functionality that is specific per product (e.g., yield in meat), and therefore the sodium reduction strategies that work in some foods may not be applicable to bouillon.

### Salt Taste and Bouillon Flavor

When a food is eaten, the flavor perceived is primarily due to a combination of taste/gustation (originating from stimulation of taste receptors on the tongue and in the mouth) and smell/olfaction (originating from the stimulation of olfactory receptors in the olfactory epithelium in the nose). The terms “taste” and “flavor” are often confused and in the current paper, the term taste refers to the technical sense (i.e., activation of taste receptors in the mouth) and the term flavor refers to the entire summed experience (from taste, smell, temperature, and other inputs) that an individual perceives when consuming a food.

#### Taste and Saltiness Detection Mechanism

The taste system includes five primary accepted taste modalities: sweet, bitter, umami (savory), salt, and sour (58). Taste is detected in the mouth via taste receptor cells located in tastebuds on the tongue. Chemicals in foods interact with specialized receptors in the taste cells, causing the taste cells to activate and send a signal directly to the brain (59). The specialized receptors vary in their structure resulting in selectivity in what food chemicals will interact with different taste receptors and thus differences in responsiveness.

The detection mechanism for each of the five primary tastes is different. The detection of bitter, sweet and umami tastes are detected by a specific set of G-coupled protein receptors (GPCR) including:

(1) Bitter taste: 25 different single GPCR (TAS2R1-48) (60–62).
(2) Sweet taste: 1 heterodimer composed of two GPCR (TAS1R2/TAS1R3) (63, 64).
(3) Umami taste: 1 heterodimer composed of two GPCR (TAS1R1/TAS1R3) (64, 65).

Bitter, sweet, and umami tastes are activated when a compound physically binds to the taste receptor that activates the taste cell and sends a signal to the brain. Salt and sour taste are initiated with the transport of salts or acids into the taste cells through ion channels, rather than activation of cell surface receptors like sweet, umami and bitter.

Salt taste is detected by ion channel receptor(s) and the exact mechanism is still not understood (66). When sodium salts, or non-sodium salts such as potassium chloride (KCl) are added to water or saliva, the salt dissociates into the positively and negatively charged ions. Only the positively charged free sodium ion is transported through ion channels in the taste cell causing the cell to be activated and transmission of a “salt” signal to the brain. Thus, it is mainly sodium that is responsible for saltiness detection and the intensity of salt an individual perceives. There are a few other positively charged ions which are transported by these ion channels and can induce a salty taste (e.g., potassium and to a lesser extent lithium and ammonium). In general, the non-sodium ions either elicit a lower salty taste, impart negative tastes (i.e., bitter/metallic) and/or are not safe for general consumption (i.e., lithium ions) (67).

The scientific literature suggests two independent mechanisms for saltiness detection. These two mechanisms are commonly referred to as amiloride sensitive and insensitive pathways due to the ability of the compound amiloride to partially suppress salt taste. Current evidence supports the epithelial sodium channel (ENaC) being responsible for amiloride sensitive salt taste detection (66). Salt taste detection through ENaC is cation selective (i.e., will only allow transport of the sodium, potassium, or lithium ions). Further, the exact mechanisms leading to salt taste following ion transport by ENaC remains unclear (68). The mechanism for amiloride insensitive salt taste detection is less understood with the cation channel responsible still to be identified. Amiloride insensitive salt taste detection can be activated by both sodium and non-sodium salts (e.g., potassium or ammonium) (66, 68, 69).

The functional mechanisms responsible for each taste (i.e., either as a G-coupled protein receptor or ion channel) have an impact on the type and range of food compounds that can activate that specific taste and determine the ability to find compounds/molecules that may function as taste substitutes. For example, the activating compounds of the sweet taste receptor are many and diverse due to the number of possible interacting sites on the sweet taste receptor (70). As the detection of saltiness is due to the transport of ions, the range of stimuli that can activate saltiness is small (e.g., restricted to the ones that can be transported *via* the salt ion channel). Therefore, due to this selectivity, there has not been and may likely not be, a true salt substitute identified (67).

Additionally, a limitation on the ability to identify salt substitutes or salt enhancers is the fact that the receptors and mechanisms involved in salt taste are still yet to be identified. For example, novel compounds imparting sweet or umami taste can be rapidly identified using screening methods with the receptors for sweet and umami. Such screening to identify new salt compounds are not possible without the identification and understanding of the salt taste receptors and cellular mechanism(s) leading to saltiness perception.

#### Human Flavor Perception

**Figure 1:**
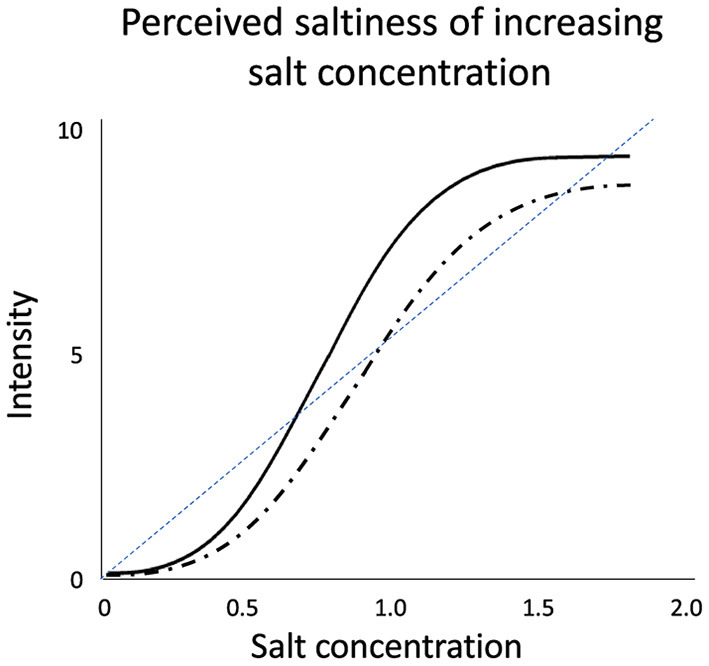
Impact of increasing salt concentration on the perceived salt intensity. Solid and dashed black lines represent theoretical psychophysical function of two consumers highlighting the variability in sensory perception between individuals. For perceived salt taste intensity, the consumer response is a sigmoid shaped response (71) and does not follow a linear pattern (dashed blue line).

Human taste response is complex. The interaction between salt concentrations within a food and perceived saltiness taste intensity does not follow a linear response (Figure 1). Salt taste perception (like other tastes and smells) has a psychophysical response that shows a sigmoid response (71). Figure 1 shows a hypothetical example of a sigmoid response for two different people (black solid and dashed lines) rating saltiness intensity to increasing concentration of a salt solution. The sigmoid response is characterized by three regions (71):

(1) Threshold region: a flat plateau region at low concentrations of sodium where no taste is perceived that leads into expansive phase resulting in exponential increase in taste,
(2) Linear phase: small increases in sodium results in a corresponding increase in perceived saltiness, and
(3) Plateau phase: compressive region at high sodium concentrations, where added sodium has little impact on increasing the perceived saltiness.

Understanding the region in which the sodium concentration in a food product is located on the sigmoid response provides a strategy for sodium reduction. For example:

(1) If a product is in the linear phase, small changes in sodium content will have a significant impact on perceived saltiness
(2) If a product is in the plateau phase, large reductions in sodium may be possible with no significant change in the perceived saltiness

Sub-saharan African bouillon has significant amounts of sodium (see Section Sodium levels in bouillon), with no published studies to date assessing bouillon saltiness in relation to this sigmoid response.

The flavor of a food is created due to a summation of individual inputs (taste, smell, and other sensory information) detected by the type and concentrations of compounds in a food. When activated in combination during eating, different tastes, and aromas can have synergistic enhancing effects on the total flavor perceived or may result in taste suppression. These interactions are referred to as taste-taste and taste-aroma interactions. These taste-taste/taste-aroma interactions can be harnessed to modify taste or flavors perceived, having the potential to enable reduction in specific ingredients (e.g., reducing salt but maintaining saltiness) or masking undesirable tastes (e.g., masking bitter off-tastes). Most of the research into taste-taste or taste-aroma interactions are completed in plain water or simple model foods. While there is evidence that the outcomes from these studies can be translated in foods, the ability to translate these directly into products is complex as salt ions will interact with different matrices depending on many factors (e.g., other flavor compounds, lipids, protein, water content, surface area) and this will affect the perception (71–73).

#### Bouillon Flavor

The original culinary concept of bouillon is essentially creating a concentrated flavourful extract or essence from cooked meat and animal products (bone marrow, protein and fat) often ameliorated with the addition of strong-tasting herbs and alliaceous vegetables (scallions, onion, garlic etc.). Bouillon normally involves roasting of components to exploit flavor, generating Maillard, and caramelization reactions. Flavors are extracted into a liquid and further concentrated into a reduction or can be formed through the mixing of raw ingredients for a dried powder or compressed cube form. The final composition is a rich mixture of flavor components, including glutamic acid, taste active umami and kokumi peptides, 5′-nucleotides (e.g., guanosine and inosine), and other molecules.

Umami represents a key component of the taste of bouillon and is activated by the amino acids L-glutamate and L-aspartic acid as well as several organic acids, umami taste enhancing compounds 5′-ribonucleotides, guanosine monophosphate (GMP) and inosine monophosphate (IMP), and peptides containing a combination of these compounds (74, 75). It can be described as a pleasant “brothy” or “meaty” taste with a long-lasting, mouth-watering and coating sensation over the tongue (76). The addition of sodium works synergistically with other components to create desirable savory and umami flavors. In addition, volatile Maillard compounds further intensify savory, meaty, and umami flavor through synergistic cross-modal interactions (77). While the presence of sodium is required for umami sensations, umami non-volatile and volatile components can strongly boost perceived saltiness in food (78). Through the judicious balance of other flavor components, the overall sodium content of bouillon cubes (or any food) can be reduced, without compromising the overall flavor intensity and consumer liking of foods.

## Key Challenges of Sodium Reduction in Bouillon for the African Market

### Taste

The flavor of a food is a key determinant of food choice and repeated use. The impact of consumer liking on food choice will generally override other factors including for example health claims or packaging (79). Salt improves the sensory properties of food (i.e., increases saltiness, enhances flavor intensity and umami taste, and masks off-tastes) and is therefore a key determinant in consumer liking. For a food company, straight reduction of sodium levels of foods can have a negative effect on overall product flavor (i.e., potential for reduced flavor impact) making it less desirable to consumers. This might be accompanied by a preference for competitor formulations with higher sodium content, or adding additional salt to food during cooking.

Another challenge for sodium reduction is due to population health initiatives that aim to fortify bouillon to overcome micronutrient deficiencies in Sub-saharan African countries (24, 38). Fortification of bouillon with micronutrients may negatively influence consumer sensory experience (e.g., iron may result in unpleasant metallic or oxidation flavors) (80). The combination of reducing salt and micronutrient fortification could accentuate these unpleasant characteristics and have greater impact on consumer acceptability. The challenge is to fortify and lower sodium levels while maintaining good flavor of the bouillon to ensure continued consumer acceptance and preference.

### Cost

A further challenge specific to Sub-saharan Africa, is to reduce sodium levels while minimally influencing the production cost and the cost for the consumer. Bouillon cubes are a low-cost staple food used in Sub-saharan African countries across all sociodemographic groups. Therefore, uptake of sodium reduction in bouillon by the food industry will only be successful if sodium reduction is completed in a manner to maintain affordability and availability to all sociodemographic groups including to low-income families. This is particularly important to ensure there is not a reduction in the frequency or amount of bouillon consumed to maximize nutritional benefit from large scale micronutrient fortification initiatives. To date, there is limited information regarding bouillon costs in Sub-saharan Africa and further the best way to cover increased costs which may be associated with sodium reduction in bouillon. Therefore, further research is required on surveys of bouillon across markets, modeling the effect of different sodium reduction strategies on bouillon cost and to understand who could cover the costs (e.g., through consumer purchase/behavior).

## Salt Reduction Strategies/Technologies for Bouillon

There are several major approaches to reducing the overall sodium content of food. With emphasis on flavor, the first is to remove sodium to a point where the consumer is unable to detect a reduction in sodium concentration in the food and the second is to replace sodium-based salts with other non-sodium salts, such as potassium chloride. The third is to exploit interactions with other taste components to enhance either the perceived saltiness and/or the overall flavor of a food. This section reviews current evidence for these sodium reduction approaches to reduce sodium in bouillon. Where there is limited research in bouillon, findings from bouillon-similar matrixes are also reviewed. The critical review of the different strategies/technologies is essential to assist both large and small bouillon producers for the Sub-saharan Africa translate research into their existing commercial products and highlight where further research is required.

It is likely that not one approach in isolation will provide the ability to significantly reduce sodium levels while maintaining the high flavor impact of bouillon. Therefore, reformulation of bouillon to lower sodium level will likely require the use of multiple strategies and technologies in combination. Additionally, many technologies and ingredients that could or are used to reduce sodium levels in the food industry are not economically viable in the Sub-saharan African market. For example, adding in higher levels of flavor from meat (i.e., chicken fat) would increase bouillon flavor, but this strategy would result in a significant increase in cost. Therefore, the strategies for sodium reduction in Sub-saharan bouillon need to balance taste, cost and suitability for use in bouillon.

### Direct Salt Removal

The simplest and cheapest method for sodium reduction is to simply remove salt from bouillon without the addition of other ingredients to counteract lower saltiness or overall flavor. However, a too large reduction in sodium will have a negative impact on consumer liking. Consumer food choices are known to be affected when the reduced sodium product does not match the characteristics of the regular sodium product (81). Therefore, for this strategy to be successful, the reduction of sodium should be less than what the average consumer is able to detect (i.e., the just noticeable difference—JND). Identification of the amount of sodium that can be reduced without consumer awareness is completed through determining difference thresholds [for recent examples in bread and meat see: (82, 83)]. If the amount of sodium is reduced in small increments less than the JND over a period of time, a degree of success can be attained without consumer awareness of the change (67). Bobowski and Vickers (84) used the difference threshold method in a series of model broths with reducing salt levels. They determined 12 steps would be required to reduce the salt content of the broth from 16.0 to 4.1 g/L, with the average sequential reduction between 10 and 20% per step without a subjects' awareness. Further assisting consumer acceptability for gradual sodium reduction is the evidence of a downward shift in salt preference over time when sodium intake is reduced across an individual's diet (85, 86).

Direct salt removal has been assessed in a range of different foods [see (87) for recent systematic review]. While there are many studies which show the ability to remove significant levels of sodium while maintaining consumer acceptance, the findings are not directly transferable to all other products. The ability to remove sodium and maintain consumer acceptance is dependent on:

(1) The matrix (liquid, semi-solid, solid) of the product which affects the spatial distribution, and release and interaction of sodium with the taste receptor,
(2) The sodium content of the product, with higher sodium products allowing greater scope for significant sodium reduction, and
(3) The contribution of the perceived saltiness to the overall flavor of the product (i.e., if saltiness is the major contributor to the flavor, the ability to reduce sodium may be reduced).

There is only one study which has assessed straight sodium reduction specifically in relation to bouillon (88). This study assessed consumer acceptance and saltiness intensity of reduced sodium bouillon (a control bouillon of 24,473 mg sodium/100 g, and three bouillons with 10, 26, and 47% lower sodium contents containing 22,025, 18,000, and 13,000 mg sodium/100 g bouillon, respectively), used in a chicken stew (final stews had 0, 4, 14, and 24% salt reduction). Chicken stews prepared with salt reduced bouillon cubes were all as equally well-liked and rated as equally salty by consumers compared to the stew containing the control bouillon with normal salt level (88). This highlights the ability for straight salt reduction as a strategy for sodium reduction in bouillon. However, the use of a stew as the matrix to assess bouillon may not be optimal to show the true impact of straight sodium reduction. Given only a single paper has assessed the impact of straight sodium reduction on the perceived saltiness and overall flavor in bouillon highlights the need for further research.

Other studies have been completed assessing sodium reduction in matrices similar to how bouillon is used, however, these studies assessed sodium reduction from salt rather than specifically from bouillon. Studies assessing soup-based matrices show the ability to remove sodium and maintain overall flavor or consumer acceptance. An overall trend is observed with the level of reduction, with a 20–33% sodium reduction associated with maintaining consumer acceptability or overall flavor (89–93). However, studies with higher sodium reduction (38–66%) resulted in a loss of consumer acceptability or overall flavor (93–95). Similarly, sodium reduction in mixed meals of different complexity, and type of matrix (solid or liquid) showed varied ability to reduce sodium. For example, consumer acceptability was maintained when sodium was reduced by a third in a soup, porridge, and beef stew but not mashed potato, while measure of saltiness was only maintained with the soup (93).

In the context of Sub-saharan African bouillon, the sodium levels are high, suggesting straight reduction for a portion of the sodium may be possible and if implemented as small steps over time provides the greatest chance to maintain consumer acceptance.

### Sodium Replacement

Sodium replacement is the process of partially substituting sodium with other mineral salts which provide a salty taste. Sodium replacement is one of the most common methods to reduce sodium content in food and beverages. Sodium replacers can include salts containing potassium, calcium, magnesium, or ammonium. As described previously (see Section Taste and saltiness detection mechanism), there is no current direct substitute for sodium, as these replacers generally impart also negative tastes (i.e., bitter/metallic), have a lower saltiness level, or are not safe for general consumption (i.e., lithium ions).

Potassium chloride is the most common sodium substitute used in food industry (96–99). Potassium is an essential nutrient already widely present in natural foods so can be added safely to processed products. An added benefit of potassium chloride is the increased potassium intake which is associated with reducing blood pressure and prevention of hypertension (99, 100). Although the physical properties of potassium salts are similar to sodium, potassium can generate undesirable bitter, metallic and acrid notes if added at high concentrations which has a negative impact on consumer acceptance (90, 96, 97). By blending mixtures of different sodium and potassium salts together, the sodium can act to naturally block bitter or off-notes of the potassium salt.

Several studies assessing the efficacy of potassium chloride substitution for sodium chloride have been completed in bouillon. These studies show the ability of potassium chloride or combination of potassium with ammonium salts to substitute 15–40% of sodium chloride while maintaining saltiness and acceptability as determined by sensory assessment using a range of both trained and untrained panelists (101, 102). However, no food matrix was used to assess the sensory impact on bouillon in these studies (i.e., the bouillon cube was diluted with water and tasted). This may overestimate the amount of potassium chloride that can be incorporated while maintaining taste, and therefore requires further research to assess in different suitable food matrices.

Taste improving agents (TIA) can also be used in combination with potassium to reduce the undesirable bitter/metallic notes. The TIAs previously studied include mineral salts, umami ingredients, food acids, amino acids, spices, simple carbohydrates, or food polymers (97). For some TIAs, the compound can both mask bitterness and contribute to enhancing salty taste (96). Most formulations combining potassium chloride and TIA are patented [see (98) for review and critical evaluation of patents in liquid savory matrices]. Of interest, the ingredients in bouillon formulation already contain many of these TIA (i.e., umami ingredients, amino acids, spices) which may result in a level of masking these off-notes, enabling use of potassium chloride as a strategy for sodium reduction. Indeed, Morris et al. (103) showed the intrinsic ability of bouillon to mask some of the potential off-flavor of potassium. Specifically, potassium chloride substitution (10%) in bouillon resulted in equal saltiness and a masking of the bitterness similar to sodium chloride control. This masking was not observed when assessed in water, emphasizing the role of TIAs (103).

Potassium chloride is already widely used by the food industry to reduce sodium levels, and on a cost basis, is one of the cheapest ingredients to enable sodium reduction in bouillon in Sub-saharan Africa. Given the high sodium salt concentration of bouillon, partial substitution with other salts may be a possible strategy for sodium reduction and should be explored as a low-cost sodium reduction strategy, particularly, in association with the other low-cost sodium reduction technologies.

### Flavor Enhancer Technologies

The concept of bouillon is to create an intense flavor addition to foods. Instinctively, cooks and chefs have been exploiting interactions between sodium, Maillard and umami molecules for optimal savory flavors and liking for a long time. With the discovery of umami in the early 1900s the complex chemistry of umami and related kokumi taste have slowly become understood by scientists (76, 104). An early insight into the umami concept, was the interaction of glutamic acid and sodium ions, which creates a savory sensation on its own and boosts overall saltiness perception. It is now apparent that other amino acids (aspartic acid, histidine), peptides, 5′-nucleotides, and related compounds work synergistically to boost flavor intensity. Additionally, novel flavor enhancing molecules are being isolated from heated and enzyme treated protein foods and Maillard reaction mixtures and reported in the scientific (105) and patent literature (106). Specific aroma volatiles are also known to enhance savory and saltiness perception (72, 107). Furthermore, some neutral non-sapid ingredients may also boost saltiness (108). The recent scientific and patent literature provide examples of technologies for sodium reduction that generally fall into three main categories; umami enhancement of savory flavor, odor induced saltiness enhancers (OISE), and mechanical salt enhancers. These are briefly summarized in the following sub sections.

#### Umami Enhancement of Savory Flavor

Compounds creating savory or umami taste include synergy between glutamic acid and sodium as well as flavor boosting 5′-nucleotides; 5′-guanosine monophosphate (5′-GMP) and 5′-inosine monophosphate (5′-IMP) (76, 78). However, umami and saltiness perception may also be enhanced by the addition of other components like free amino acids (97, 109–111), small peptides (74, 112), protein hydrolysates (113, 114) and other molecules isolated from Maillard reactions (115, 116). Additionally, the chemical structure of 5′-nucleotides has been used as a scaffold to synthesize novel potent taste enhancers with similar sensory profiles as MSG (115). The ability of umami compounds to enable reduced sodium levels in foods is due to either the enhancement of the overall flavor (due to heightened umami/savory taste) or due to synergistic interactions between umami compounds and salt to boost saltiness perception (taste-taste interactions).

The primary role of glutamic acid in enhancing savory and saltiness perception has been well-known for decades. Glutamic acid occurs in high abundance naturally in many foods (e.g., aged cheese and meat, dried seaweed, and tomatoes). Glutamate is also added to many processed foods in the form of its sodium salt, MSG. The optimum pleasantness concentration of glutamate, as a flavor enhancer of food, has been established to be between 0.2 and 0.8 %w/w depending on the food matrix (117, 118). European Commission Regulation No. 1129/2011 sets a maximum limit of 10 g/kg of MSG and its salts in food products, except for non-processed foods and seasoning which have no specific maximum (119).

Similarly, 5′-GMP and 5′-IMP in the presence of glutamate are widely known to boost umami character and saltiness perception. The optimum ratio of MSG to these nucleotides is usually between 100:1 and 50:1 (120). However, the exact optimum depends on the food matrix and other ingredients. MSG, 5′-GMP and 5′-IMP are already widely used ingredients in bouillon products at relatively high concentrations. Hence it is expected that ideal quantities and optimal ratios of these ingredients in bouillon formulations are well-understood and utilized by manufacturers. Therefore, there is likely limited opportunities to significantly reduce sodium levels through increased addition of umami compounds.

Enzyme protein hydrolysates (soy and other protein) and dried mushroom and tomato powders are naturally rich in umami free amino acids and small peptides which can boost savory and overall flavor of a food (114, 121, 122). Yeast extracts are rich in 5′-nucleotides and are also widely used to boost saltiness perception and umami. In addition, organic acids such as succinic and citric acid are known to enhance the perceived saltiness of foods without increasing sourness (123). An extensive list of high umami foods and molecules are available at Umami Information Center (https://www.umamiinfo.com/umamidb/). While these ingredients continue to be explored in markets where there is a desire for “clean label” ingredients and minimally processed foods, they are expensive alternatives to MSG. Until those ingredients can be sourced and processed at minimal cost, it is expected that enzyme hydrolysates and powders of natural foods won't be suitable alternatives in the production of affordable reduced sodium bouillon.

#### Odor Induced Saltiness Enhancers

There is significant research on the cross-modal interaction between odors and aroma (olfactory receptor stimulants) and taste (saltiness and sweetness perception) (72, 124). Salt-congruent or savory-congruent odors are most effective in enhancing perceived saltiness; for example, a high degree of OISE was achieved with aroma extracts from bacon, anchovy and ham added to salt solutions (125, 126). The aroma of fermented soybean significantly increased the perceived saltiness of snack foods (127, 128). Similarly, sulfur volatiles from alliaceous vegetables and extracts from herbs (129, 130) and celery (a natural source of nitrates) can boost saltiness perception (131, 132). Not only whole aroma extracts from food have been evaluated for their salt boosting effects, but also single salt-congruent volatiles. For example, the volatile lactone compound sotolone significantly boosted perceived saltiness in solution, allowing up to 30% sodium reduction (102). Additional evidence suggests for OISE is optimal for a product containing low to moderate saltiness level rather than a high saltiness level (133). It is likely that an OISE approach is used in some current sodium reduced commercial bouillon products. In summary, targeted salt-congruent whole food odor extracts and single odor components can be optimized to enhance saltiness perception in food and potentially in bouillon.

#### Mechanical Salt Enhancers

Mechanical salt enhancers (MSE) refer to food ingredients which change the overall microstructure of the food matrix, increasing either the availability of sodium ions in solution or the localized sodium ion concentration near sodium receptors on the tongue. A recent study showed that addition of the slightly acidic polysaccharide gum Arabic (GA, 0.1 and 0.3%) enhanced the saltiness perception of salt solution by up to 30% (134). The authors proposed that the gum Arabic increased “mucopenetration” on the tongue surface and saltiness perception. Psyllium husk gum is mentioned as a component in a salt boosting bouillon formulation described in a recent patent by Unilever (135).

Chitin is one of the most abundant biopolymers in nature, present in arthropod exoskeletons fungi and cell walls. Normally chitin is only sparingly soluble in aqueous solution. Researchers recently produced chitin nanoparticles using an ultrasound cell grinder, increasing the water solubility (123). The authors increased the sensory saltiness rating of fish filets soaked in a brine of citric acid, salt and chitin fibers (0.15 g/L). The results suggests that mechanical salt enhancers like gum Arabic or chitin at low concentrations in a bouillon broth may increase the availability of sodium ions and perceived saltiness, although this would have to be experimentally determined.

### Technology to Redistribute Salt/Alter Salt Release

The versatility of bouillon use is the key challenge for sodium reduction and is a unique challenge to bouillon compared to other processed foods. Firstly, bouillon undergoes changes in structure, starting off as a dry cube and generally undergoing transformation in liquid matrix or spread through a solid matrix. Secondly, bouillon has a diverse range of uses and can be added to any variety of savory dishes. This could include:

(1) Dissolving and use as a liquid stock, i.e., diluted in water then added to food or added directly to a liquid matrix (soup/stew),
(2) Crushed and added to food while cooking, e.g., coated over meat as a dry rub/marinade, or
(3) Crushed and sprinkled over food after cooking.

Additionally, bouillon cubes can be stored for long periods of time potentially in humid conditions, and when used, they are exposed to extreme conditions during preparation of a meal (i.e., exposure to heat/boiling/pressure/acid for prolonged periods).

Thus, technologies employed for sodium reduction in bouillon need to consider these factors and are different to other processed foods where sodium reduction technologies are “set” within the product and are maintained until consumed. Therefore, strategies/technologies commonly used for sodium reduction in these other processed foods (i.e., breads or processed meats) are not suitable for use in bouillon cubes. Examples include, manipulating salt crystal size (136) or altering the distribution of sodium in a product (137).

#### The Matrix/Vehicle to Assess Bouillon

A key area that is not well-defined is the correct matrices/meal format to use in sensory analyses to assess sodium reduction technologies in bouillon. The matrix that is used in the sensory analysis (for example as a liquid broth or in a stew) will have a large impact on the ability and magnitude for a sodium reduction method to reduce sodium levels while retaining flavor (138). The research conducted to date on bouillon has used either straight bouillon cube reconstituted with water following manufacturer's directions (80, 101, 102, 139, 140) or a meat- or vegetable-based stew (complex dish with lots of added other flavors) (80, 88, 93). These might not represent optimal matrices to assess bouillon as:

(1) The reconstituted bouillon represents a strongly concentrated form of the product which is not representative of how the product is normally consumed (i.e., maybe located at the high plateau region, see Section Human flavor perception and Figure 1), or
(2) The bouillon only contributes a small component of the overall flavor in a stew or complex meal.

In both these instances, the matrix selected may overestimate the possibility for sodium reduction (while maintaining consumer acceptance). Therefore, prior to assessing different sodium reduction technologies in bouillon, there is a need to test and define the optimal matrices for sensory analysis to ensure research findings can be translated by industry into their products.

### Masking Negative Flavors due to Fortification

Bouillon is receiving increasing attention as a fortification vehicle for delivery of micronutrients in Sub-saharan Africa (24, 141, 142). Fortification of bouillon with micronutrients may result in changing the organoleptic properties that may reduce consumer acceptability (i.e., undesirable changes in taste or visual look of the food product). Especially when iron is added to the product, the taste, appearance and shelf life can be affected (80). For example, some iron fortificants may cause a metallic flavor as well-oxidative/rancid characteristics. Additionally, iron can affect the stability of other micronutrients (e.g., vitamin A and C or iodine) and during storage the undesirable characteristics can accentuate. The presence of undesirable characteristics is dependent on the iron source, with current iron fortification in commercial bouillon using ferric pyrophosphate (FePP), which has a low impact on organoleptic properties, however FePP has a low rate of absorption by the body (low bio-availability) compared to other iron fortificants (38).

Flavor intensity may be lower in salt reduced products and still accepted by the consumer, but in this frame, off-flavors or other organoleptic changes may become more obvious due to added fortificants. As efforts for bouillon fortification increase, there is a need to understand when the possible organoleptic changes occur between the matrix and the fortified nutrients. Therefore, understanding and integrating information on volatile and non-volatile composition of bouillon will allow to identify possible flavor enhancers, ingredients, or technologies to either mask or reduce the effect of fortification, in addition to potential flavor loss due to reducing sodium levels of bouillon.

## Conclusion

The work presents an overview of the salt reduction strategies with focus on Bouillon cubes. Bouillon cubes provide flavor enhancement to savory foods and contain high levels of sodium. Bouillon consumption in Sub-saharan African countries is high, with daily consumption across all sociodemographic groups. This has resulted in efforts to fortify bouillon to overcome micronutrient deficiencies. However, there is growing evidence that bouillon consumption is a contributor to sodium intake in Sub-saharan African countries above recommended levels and further research is required to better quantify this contribution. Therefore, population level health effects to reduce NCD could be achieved through the reduction of sodium levels in commercial bouillon products in Sub-saharan African countries. While there is currently not a single low-cost ingredient to replace salt having the same taste and flavor profile, reformulations of bouillon using multiple low-cost strategies are a viable solution to enable significant sodium reduction resulting in population wide health outcomes to reduce hypertension and CVD in Sub-saharan Africa.

## Author Contributions

NA: conceptualization, writing—original draft, writing—review and editing, and project administration. MC-B, MM, and GG-B: writing—original draft and writing—review and editing. PL-S and LL: conceptualization and writing—review and editing. DF: conceptualization, writing—original draft, and writing—review and editing. All authors contributed to the article and approved the submitted version.

## Funding

This work was supported by a grant from the Bill and Melinda Gates Foundation under Grant INV-002988.

## Conflict of Interest

The authors declare that the research was conducted in the absence of any commercial or financial relationships that could be construed as a potential conflict of interest.

## Publisher's Note

All claims expressed in this article are solely those of the authors and do not necessarily represent those of their affiliated organizations, or those of the publisher, the editors and the reviewers. Any product that may be evaluated in this article, or claim that may be made by its manufacturer, is not guaranteed or endorsed by the publisher.

## References

[1] Powles J Fahimi S Micha R Khatibzadeh S Shi P Ezzati M. Global, regional and national sodium intakes in 1990 and 2010: a systematic analysis of 24 h urinary sodium excretion and dietary surveys worldwide. BMJ Open. (2013) 3:e003733. 10.1136/bmjopen-2013-003733 24366578 PMC3884590

[2] Thout SR Santos JA McKenzie B Trieu K Johnson C McLean R. The science of salt: updating the evidence on global estimates of salt intake. J Clin Hypertens. (2019) 21:710–21. 10.1111/jch.13546 31033166 PMC8030574

[3] Oyebode O Oti S Chen Y-F Lilford RJ. Salt intakes in sub-Saharan Africa: a systematic review and meta-regression. Popul Health Metr. (2016) 14:1. 10.1186/s12963-015-0068-7 26759530 PMC4709973

[4] Mizéhoun-Adissoda C Houinato D Houehanou C Chianea T Dalmay F Bigot A. Dietary sodium and potassium intakes: data from urban and rural areas. Nutrition. (2017) 33:35–41. 10.1016/j.nut.2016.08.007 27908548

[5] Melse-Boonstra A Rozendaal M Rexwinkel H Gerichhausen MJ van den Briel T Bulux J. Determination of discretionary salt intake in rural Guatemala and Benin to determine the iodine fortification of salt required to control iodine deficiency disorders: studies using lithium-labeled salt. Am J Clin Nutr. (1998) 68:636–41. 10.1093/ajcn/68.3.636 9734741

[6] Queiroz A Damasceno A Jessen N Novela C Moreira P Lunet N. Urinary sodium and potassium excretion and dietary sources of sodium in Maputo, Mozambique. Nutrients. (2017) 9:830. 10.3390/nu9080830 28771193 PMC5579623

[7] Alves D Santos Z Amado M Craveiro I Delgado AP Correia A. Low potassium and high sodium intakes: a double health threat to Cape Verdeans. BMC Public Health. (2018) 18:995. 10.1186/s12889-018-5911-x 30092771 PMC6085717

[8] Prynn JE Banda L Amberbir A Price AJ Kayuni N Jaffar S. Dietary sodium intake in urban and rural Malawi, and directions for future interventions. Am J Clin Nutr. (2018) 108:587–93. 10.1093/ajcn/nqy125 29982267 PMC6134286

[9] Menyanu E Baatiema L Charlton K Wilson M Aikins AD-G Russell J. Towards population salt reduction to control high blood pressure in Ghana: a policy direction. Curr Dev Nutr. (2020) 4(Supplement_3) 2020:nzaa084. 10.1093/cdn/nzaa084 32851200 PMC7438700

[10] Kearney J. Food consumption trends and drivers. Philos Trans R Soc B Biol Sci. (2010) 365:2793–807. 10.1098/rstb.2010.0149 20713385 PMC2935122

[11] Popkin BM Adair LS Ng SW. Global nutrition transition and the pandemic of obesity in developing countries. Nutr Rev. (2012) 70:3–21. 10.1111/j.1753-4887.2011.00456.x 22221213 PMC3257829

[12] Strazzullo P D'Elia L Kandala N-B Cappuccio FP. Salt intake, stroke, and cardiovascular disease: meta-analysis of prospective studies. BMJ. (2009) 339:b4567. 10.1136/bmj.b4567 19934192 PMC2782060

[13] Forouzanfar MH Afshin A Alexander LT Anderson HR Bhutta ZA Biryukov S. Global, regional, and national comparative risk assessment of 79 behavioural, environmental and occupational, and metabolic risks or clusters of risks, 1990–2015: a systematic analysis for the global burden of disease study 2015. Lancet. (2016) 388:1659–724. 10.1016/S0140-6736(17)32366-8 27733284 PMC5388856

[14] Mozaffarian D Fahimi S Singh GM Micha R Khatibzadeh S Engell RE. Global sodium consumption and death from cardiovascular causes. N Engl J Med. (2014) 371:624–34. 10.1056/NEJMoa1304127 25119608

[15] He FJ Li J MacGregor GA. Effect of longer term modest salt reduction on blood pressure: cochrane systematic review and meta-analysis of randomised trials. BMJ. (2013) 346:f1325. 10.1136/bmj.f1325 23558162

[16] Menyanu E Charlton KE Ware LJ Russell J Biritwum R Kowal P. Salt use behaviours of Ghanaians and South Africans: a comparative study of knowledge, attitudes and practices. Nutrients. (2017) 9:939. 10.3390/nu9090939 28846641 PMC5622699

[17] World Health Organization. A Global Brief on Hypertension. Silent Killer, Global Public Health Crisis. Geneva: World Health Organization (2013). 10.5005/ijopmr-24-1-2

[18] World Health Organization. Global Status Report on Noncommunicable Diseases 2010. Geneva: WHO (2011).

[19] Mills KT Stefanescu A He J. The global epidemiology of hypertension. Nat Rev Nephrol. (2020) 16:223–37. 10.1038/s41581-019-0244-2 32024986 PMC7998524

[20] Ataklte F Erqou S Kaptoge S Taye B Echouffo-Tcheugui JB Kengne AP. Burden of undiagnosed hypertension in Sub-Saharan Africa. Hypertension. (2015) 65:291–8. 10.1161/HYPERTENSIONAHA.114.04394 25385758

[21] Opie LH Seedat YK. Hypertension in Sub-Saharan African populations. Circulation. (2005) 112:3562–8. 10.1161/CIRCULATIONAHA.105.539569 16330697

[22] World Health Organization. Health in 2015: From MDGs to SDGs. Geneva: WHO (2015).

[23] Blüthner A Vierck L. Setting standards for business and development: how legal frameworks can support market-based nutrition partnerships. Eur Food Feed Law Rev. (2009) 4:104–18.

[24] Mkambula P Mbuya MNN Rowe LA Sablah M Friesen VM Chadha M. The unfinished agenda for food fortification in low- and middle-income countries: quantifying progress, gaps and potential opportunities. Nutrients. (2020) 12:354. 10.3390/nu12020354 32013129 PMC7071326

[25] World Health Organization. Tackling NCDs: 'Best Buys' and Other Recommended Interventions for the Prevention and Control of Noncommunicable Diseases. Geneva: World Health Organization (2017).

[26] World Health Organization. SHAKE the Salt Habit. The SHAKE Technical Package for Salt Reduction. Geneva: World Health Organization (2016).

[27] World Health Organization. Global Action Plan for the Prevention and Control of NCDs 2013–2020. WHO (2013).

[28] Tekle DY Santos JA Trieu K Thout SR Ndanuko R Charlton K. Monitoring and implementation of salt reduction initiatives in Africa: a systematic review. J Clin Hypertens. (2020) 22:1355–70. 10.1111/jch.13937 32770701 PMC7496579

[29] Watkins DA Olson ZD Verguet S Nugent RA Jamison DT. Cardiovascular disease and impoverishment averted due to a salt reduction policy in South Africa: an extended cost-effectiveness analysis. Health Policy Plan. (2016) 31:75–82. 10.1093/heapol/czv023 25841771 PMC4724166

[30] Aminde LN Cobiac LJ Veerman JL. Potential impact of a modest reduction in salt intake on blood pressure, cardiovascular disease burden and premature mortality: a modelling study. Open Heart. (2019) 6:e000943. 10.1136/openhrt-2018-000943 30997132 PMC6443119

[31] Wamba AA Takah NF Johnman C. The impact of interventions for the primary prevention of hypertension in Sub-Saharan Africa: A systematic review and meta-analysis. PLoS ONE. (2019) 14:e0219623. 10.1371/journal.pone.0219623 31323041 PMC6641142

[32] Muthuri SK Oti SO Lilford RJ Oyebode O. Salt reduction interventions in Sub-Saharan Africa: a systematic review. PLoS ONE. (2016) 11:e0149680. 10.1371/journal.pone.0149680 26963805 PMC4786148

[33] Farquhar WB Edwards DG Jurkovitz CT Weintraub WS. Dietary sodium and health: more than just blood pressure. J Am Coll Cardiol. (2015) 65:1042–50. 10.1016/j.jacc.2014.12.039 25766952 PMC5098396

[34] Kloss L Meyer JD Graeve L Vetter W. Sodium intake and its reduction by food reformulation in the European union — a review. NFS J. (2015) 1:9–19. 10.1016/j.nfs.2015.03.001

[35] Leyvraz M Mizéhoun-Adissoda C Houinato D Moussa Baldé N Damasceno A Viswanathan B. Food consumption, knowledge, attitudes, and practices related to salt in urban areas in five Sub-Saharan African countries. Nutrients. (2018) 10:1028. 10.3390/nu10081028 30087242 PMC6116014

[36] Engle-Stone R Ndjebayi AO Nankap M Brown KH. Consumption of potentially fortifiable foods by women and young children varies by ecological zone and socio-economic status in Cameroon. J Nutr. (2012) 142:555–65. 10.3945/jn.111.148783 22323765

[37] Hess SY Brown KH Sablah M Engle-Stone R Aaron GJ Baker SK. Results of fortification rapid assessment tool (FRAT) surveys in Sub-Saharan Africa and suggestions for future modifications of the survey instrument. Food Nutr Bull. (2013) 34:21–38. 10.1177/156482651303400104 23767278

[38] Moretti D Hurrell RF Cercamondi CI. Chapter 16 - bouillon cubes. In: Mannar MGV Hurrell RF editors. Food Fortification in a Globalized World. London: Academic Press (2018). p. 159–65. 10.1016/B978-0-12-802861-2.00016-X

[39] Abizari A-R Dold S Kupka R Zimmermann MB. More than two-thirds of dietary iodine in children in northern Ghana is obtained from bouillon cubes containing iodized salt. Public Health Nutr. (2017) 20:1107–13. 10.1017/S1368980016003098 27903312 PMC10261352

[40] Chen Z Oldewage-Theron W. Household consumption of stock cubes and stock powder in the Vaal Triangle of SA. Nutr Food Sci. (2004) 34:174–8. 10.1108/00346650410544873

[41] Adeyemo A Prewitt T Luke A Omotade O Rotimi C Brieger W. The feasibility of implementing a dietary sodium reduction intervention among free-living normotensive individuals in south West Nigeria. Ethn Dis. (2002) 12:207–12. 12019929

[42] Mezue K. The increasing burden of hypertension in Nigeria – can a dietary salt reduction strategy change the trend? Perspect Public Health. (2013) 134:346–52. 10.1177/1757913913499658 24002906

[43] Charlton KE Steyn K Levitt NS Peer N Jonathan D Gogela T. A food-based dietary strategy lowers blood pressure in a low socio-economic setting: a randomised study in South Africa. Public Health Nutr. (2008) 11:1397–406. 10.1017/S136898000800342X 18752692

[44] Vasconcelos AM Santos SML Damaceno MN Cavalcante ABD. Physicochemical characterization and comparison of labels of beef bouillon cubes. Food Sci Technol. (2018) 38:639–42. 10.1590/fst.11017

[45] Adjangba K Agbodji Y Agoro S Amouzou E. Cooking salt content in food cubes commonly used in Northern Togo. Open J Nutr Food Sci. (2020) 2.

[46] Spohrer R Knowles J Jallier V Ndiaye B Indorf C Guinot P. Estimation of population iodine intake from iodized salt consumed through bouillon seasoning in Senegal. Ann N Y Acad Sci. (2015) 1357:43–52. 10.1111/nyas.12963 26767583

[47] Peters SAE Dunford E Ware LJ Harris T Walker A Wicks M. The sodium content of processed foods in south africa during the introduction of mandatory sodium limits. Nutrients. (2017) 9:404. 10.3390/nu9040404 28425938 PMC5409743

[48] World Health Organization. Intersectorial Case Study: Successful Sodium Regulation in South Africa (2013).

[49] Legetic B Campbell N. Reducing salt intake in the Americas: pan American health organization actions. J Health Commun. (2011) 16:37–48. 10.1080/10810730.2011.601227 21916712

[50] Allemandi L Tiscornia MV Guarnieri L Castronuovo L Martins E. Monitoring sodium content in processed foods in argentina 2017–2018: compliance with national legislation and regional targets. Nutrients. (2019) 11:1474. 10.3390/nu11071474 31261665 PMC6682874

[51] Public Health England. Salt Reduction Targets for 2017. (2017). Available online at: https://www.gov.uk/government/publications/salt-reduction-targets-for-20172017

[52] Dötsch-Klerk M Pmm Goossens W Meijer GW van het Hof KH. Reducing salt in food; setting product-specific criteria aiming at a salt intake of 5 g per day. Eur J Clin Nutr. (2015) 69:799–804. 10.1038/ejcn.2015.5 25690867 PMC4493648

[53] Nijman CAJ Zijp IM Sierksma A Roodenburg AJC Leenen R van den Kerkhoff C. A method to improve the nutritional quality of foods and beverages based on dietary recommendations. Eur J Clin Nutr. (2007) 61:461–71. 10.1038/sj.ejcn.1602548 17119547

[54] Vlassopoulos A Masset G Charles VR Hoover C Chesneau-Guillemont C Leroy F. A nutrient profiling system for the (re)formulation of a global food and beverage portfolio. Eur J Nutr. (2017) 56:1105–22. 10.1007/s00394-016-1161-9 26879847 PMC5346408

[55] Vieux F Privet L Masset G. Food- and diet-based validations of a nestlé nutrient profiling system for reformulation in two nationally representative surveys. Br J Nutr. (2018) 120:1056–64. 10.1017/S0007114518002490 30355394

[56] Hayabuchi H Morita R Ohta M Nanri A Matsumoto H Fujitani S. Validation of preferred salt concentration in soup based on a randomized blinded experiment in multiple regions in Japan-influence of umami (L-glutamate) on saltiness and palatability of low-salt solutions. Hypertens Res. (2020) 43:525–33. 10.1038/s41440-020-0397-1 31996813 PMC8075858

[57] Maluly HDB Arisseto-Bragotto AP Reyes FGR. Monosodium glutamate as a tool to reduce sodium in foodstuffs: technological and safety aspects. Food Sci Nutr. (2017) 5:1039–48. 10.1002/fsn3.499 29188030 PMC5694874

[58] Chandrashekar J Hoon MA Ryba NJP Zuker CS. The receptors and cells for mammalian taste. Nature. (2006) 444:288–94. 10.1038/nature05401 17108952

[59] Chaudhari N Roper SD. The cell biology of taste. J Cell Biol. (2010) 190:285–96. 10.1083/jcb.201003144 20696704 PMC2922655

[60] Adler E Hoon MA Mueller KL Chandrashekar J Ryba NJP Zuker CS. A novel family of mammalian taste receptors. Cell. (2000) 100:693–702. 10.1016/S0092-8674(00)80705-9 10761934

[61] Behrens M Foerster S Staehler F Raguse J-D Meyerhof W. Gustatory expression pattern of the human TAS2R bitter receptor gene family reveals a heterogenous population of bitter responsive taste receptor cells. J Neurosci. (2007) 27:12630–40. 10.1523/JNEUROSCI.1168-07.2007 18003842 PMC6673335

[62] Chandrashekar J Mueller KL Hoon MA Adler E Feng L Guo W. T2Rs function as bitter taste receptors. Cell. (2000) 100:703–11. 10.1016/S0092-8674(00)80706-0 10761935

[63] Nelson G Hoon MA Chandrashekar J Zhang Y Ryba NJP Zuker CS. Mammalian sweet taste receptors. Cell. (2001) 106:381–90. 10.1016/S0092-8674(01)00451-2 11509186

[64] Zhao GQ Zhang Y Hoon MA Chandrashekar J Erlenbach I Ryba NJP. The receptors for mammalian sweet and umami taste. Cell. (2003) 115:255–66. 10.1016/S0092-8674(03)00844-4 14636554

[65] Nelson G Chandrashekar J Hoon MA Feng L Zhao G Ryba NJP. An amino-acid taste receptor. Nature. (2002) 416:199–202. 10.1038/nature726 11894099

[66] Roper SD. The taste of table salt. Eur J Physiol. (2015) 467:457–63. 10.1007/s00424-014-1683-z 25559847 PMC4326615

[67] Henney JE. Taste and Flavor Roles of Sodium in Foods: A Unique Challenge to Reducing Sodium Intake. In: Henney JE Taylor CL Boon CS editors. Strategies to Reduce Sodium Intake in the United States. Washington, DC: The National Academies Press (2010). 10.17226/12818 21210559

[68] Bachmanov AA Bosak NP Lin C Matsumoto I Ohmoto M Reed DR. Genetics of taste receptors. Curr Pharm Des. (2014) 20:2669–83. 10.2174/13816128113199990566 23886383 PMC4764331

[69] Lewandowski BC Sukumaran SK Margolskee RF Bachmanov AA. Amiloride-insensitive salt taste is mediated by two populations of type III taste cells with distinct transduction mechanisms. J Neurosci. (2016) 36:1942–53. 10.1523/JNEUROSCI.2947-15.2016 26865617 PMC4748077

[70] Behrens M Briand L de March CA Matsunami H Yamashita A Meyerhof W. Structure–Function relationships of olfactory and taste receptors. Chem Senses. (2018) 43:81–7. 10.1093/chemse/bjx083 29342245 PMC6276892

[71] Keast RSJ Breslin PAS. An overview of binary taste–taste interactions. Food Qual Prefer. (2003) 14:111–24. 10.1016/S0950-3293(02)00110-6

[72] Thomas-Danguin T Guichard E Salles C. Cross-modal interactions as a strategy to enhance salty taste and to maintain liking of low-salt food: a review. Food Funct. (2019) 10:5269–81. 10.1039/C8FO02006J 31436262

[73] Mojet J Heidema J Christ-Hazelhof E. Effect of concentration on taste–taste interactions in foods for elderly and young subjects. Chem Senses. (2004) 29:671–81. 10.1093/chemse/bjh070 15466812

[74] Zhang Y Venkitasamy C Pan Z Liu W Zhao L. Novel umami ingredients: umami peptides and their taste. J Food Sci. (2017) 82:16–23. 10.1111/1750-3841.13576 27926796

[75] Suess B Festring D Hofmann T. 15—Umami compounds and taste enhancers. In: Parker JK Elmore JS Methven L editors. Flavour Development, Analysis and Perception in Food and Beverages. Cambridge, UK: Woodhead Publishing (2015). p. 331–51. 10.1016/B978-1-78242-103-0.00015-1

[76] Kurihara K. Umami the fifth basic taste: history of studies on receptor mechanisms and role as a food flavor. Biomed Res Int. (2015) 2015:189402. 10.1155/2015/189402 26247011 PMC4515277

[77] Jayasena DD Ahn DU Nam KC Jo C. Flavour chemistry of chicken meat: a review. Asian-Australas J Anim Sci. (2013) 26:732–42. 10.5713/ajas.2012.12619 25049846 PMC4093335

[78] Rocha RAR Ribeiro MN Silva GA Rocha LCR Pinheiro ACM Nunes CA. Temporal profile of flavor enhancers MAG, MSG, GMP, and IMP, and their ability to enhance salty taste, in different reductions of sodium chloride. J Food Sci. (2020) 85:1565–75. 10.1111/1750-3841.15121 32282071

[79] Lima M de Alcantara M Ares G Deliza R. It is not all about information! Sensory experience overrides the impact of nutrition information on consumers' choice of sugar-reduced drinks. Food Qual Prefer. (2019) 74:1–9. 10.1016/j.foodqual.2018.12.013

[80] Klassen-Wigger P Geraets M Messier MC Detzel P Lenoble HP Barclay DV. Chapter 39 - micronutrient fortification of bouillon cubes in central and west africa. In: Mannar MGV Hurrell RF editors. Food Fortification in a Globalized World. London: Academic Press (2018). p. 363–72. 10.1016/B978-0-12-802861-2.00039-0

[81] Zandstra EH Lion R Newson RS. Salt reduction: moving from consumer awareness to action. Food Qual Prefer. (2016) 48:376–81. 10.1016/j.foodqual.2015.03.005

[82] Antúnez L Giménez A Ares G. A consumer-based approach to salt reduction: case study with bread. Food Res Int. (2016) 90:66–72. 10.1016/j.foodres.2016.10.015 29195892

[83] Cubero-Castillo E Araya-Morice A Hernandez-Campos D Araya-Quesada Y. Salt reduction without consumer awareness using a sensory threshold approach: a case study in meat products. CyTA J Food. (2019) 17:763–9. 10.1080/19476337.2019.1648556

[84] Bobowski N Vickers Z. Determining sequential difference thresholds for sodium chloride reduction. J Sens Stud. (2012) 27:168–75. 10.1111/j.1745-459X.2012.00379.x

[85] Bertino M Beauchamp GK Engelman K. Long-term reduction in dietary sodium alters the taste of salt. Am J Clin Nutr. (1982) 36:1134–44. 10.1093/ajcn/36.6.1134 7148734

[86] Blais CA Pangborn RM Borhani NO Ferrell MF Prineas RJ Laing B. Effect of dietary sodium restriction on taste responses to sodium chloride: a longitudinal study. Am J Clin Nutr. (1986) 44:232–43. 10.1093/ajcn/44.2.232 3728360

[87] Jaenke R Barzi F McMahon E Webster J Brimblecombe J. Consumer acceptance of reformulated food products: a systematic review and meta-analysis of salt-reduced foods. Crit Rev Food Sci Nutr. (2017) 57:3357–72. 10.1080/10408398.2015.1118009 26745848

[88] De Kock HL Zandstra EH Sayed N Wentzel-Viljoen E. Liking, salt taste perception and use of table salt when consuming reduced-salt chicken stews in light of South Africa's new salt regulations. Appetite. (2016) 96:383–90. 10.1016/j.appet.2015.09.026 26415915

[89] Willems AA van Hout DHA Zijlstra N Zandstra EH. Effects of salt labelling and repeated in-home consumption on long-term liking of reduced-salt soups. Public Health Nutr. (2014) 17:1130–7. 10.1017/S1368980013001055 23635386 PMC10282450

[90] Lee CL Lee SM Kim K-O. Use of consumer acceptability as a tool to determine the level of sodium reduction: a case study on beef soup substituted with potassium chloride and soy-sauce odor. J Food Sci. (2015) 80:S2570–S7. 10.1111/1750-3841.13098 26447813

[91] Leong J Kasamatsu C Ong E Hoi JT Loong MN. A study on sensory properties of sodium reduction and replacement in Asian food using difference-from - control test. Food Sci Nutr. (2016) 4:469–78. 10.1002/fsn3.308 27247776 PMC4867766

[92] Methven L Langreney E Prescott J. Changes in liking for a no added salt soup as a function of exposure. Food Qual Prefer. (2012) 26:135–40. 10.1016/j.foodqual.2012.04.012

[93] Malherbe M Walsh C Merwe C. Consumer acceptability and salt perception of food with a reduced sodium content. J Consum Sci. (2003) 31:12–21. 10.4314/jfecs.v31i1.52833

[94] Mitchell M Brunton NP Wilkinson MG. The influence of salt taste threshold on acceptability and purchase intent of reformulated reduced sodium vegetable soups. Food Qual Prefer. (2013) 28:356–60. 10.1016/j.foodqual.2012.11.002

[95] Nguyen H Wismer WV. A comparison of sensory attribute profiles and liking between regular and sodium-reduced food products. Food Res Int. (2019) 123:631–41. 10.1016/j.foodres.2019.05.037 31285012

[96] Ben Abu N Harries D Voet H Niv MY. The taste of KCl - what a difference a sugar makes. Food Chem. (2018) 255:165–73. 10.1016/j.foodchem.2018.01.175 29571463

[97] Akgun B Genc S Cheng Q Isik O. Impacts of sodium chloride reduction in tomato soup system using potassium chloride and amino acids. Czech J Food Sci. (2019) 37:93–8. 10.17221/140/2018-CJFS140

[98] Cepanec K Vugrinec S Cvetkovic T Ranilovic J. Potassium chloride-based salt substitutes: a critical review with a focus on the patent literature. Compre Rev Food Sci Food Saf. (2017) 16:881–94. 10.1111/1541-4337.12291 33371617

[99] Van Buren L Dötsch-Klerk M Seewi G Newson RS. Dietary impact of adding potassium chloride to foods as a sodium reduction technique. Nutrients. (2016) 8:235. 10.3390/nu8040235 27110818 PMC4848703

[100] Geleijnse JM Kok FJ Grobbee DE. Blood pressure response to changes in sodium and potassium intake: a metaregression analysis of randomised trials. J Hum Hypertens. (2003) 17:471–80. 10.1038/sj.jhh.1001575 12821954

[101] Askar A Elsamahy SK Shehata HA Tawfik M. Pasterma and beef bouillon - the effect of substituting KCl and K-lactate for sodium chloride. Fleischwirtschaft. (1993) 73:289–92.

[102] Batenburg M van der Velden R. Saltiness enhancement by savory aroma compounds. J Food Sci. (2011) 76:S280–8. 10.1111/j.1750-3841.2011.02198.x 22417442

[103] Morris C Labarre C Koliandris AL Hewson L Wolf B Taylor AJ. Effect of pulsed delivery and bouillon base on saltiness and bitterness perceptions of salt delivery profiles partially substituted with KCl. Food Qual Prefer. (2010) 21:489–94. 10.1016/j.foodqual.2010.01.002

[104] Yang J Bai W Zeng X Cui C. Gamma glutamyl peptides: the food source, enzymatic synthesis, kokumi-active and the potential functional properties – a review. Trends Food Sci Technol. (2019) 91:339–46. 10.1016/j.tifs.2019.07.022

[105] Hong J-H Kwon K-Y Kim K-O. Sensory characteristics and consumer acceptability of beef stock containing the glutathione-xylose maillard reaction product and/or monosodium glutamate. J Food Sci. (2012) 77:S233–9. 10.1111/j.1750-3841.2012.02724.x 22591448

[106] Smarrito-Menozzi CM Barcos ME Viton F Manganiello S Nestec SA. Sugar-Dipeptide Conjugates as Flavor Molecules Patent WO2018/015413Al (2017).

[107] Thomas-Danguin T Lawrence G Emorine M Nasri N Boisard L Guichard E. Strategies to enhance saltiness in food involving cross modal interactions. In: Guthrie B Beauchamp J Buettner A Lavine BK editors. Chemical Sensory Informatics of Food: Measurement, Analysis, Integration. Washington DC: Oxford University Press (2015). p. 27–40. 10.1021/bk-2015-1191.ch003

[108] Soldo T Blank I Hofmann T. (+)-(S)-alapyridaine—a general taste enhancer? Chem Senses. (2003) 28:371–9. 10.1093/chemse/28.5.371 12826533

[109] Ghirri A Bignetti E. Occurrence and role of umami molecules in foods. Int J Food Sci Nutr. (2012) 63:871–81. 10.3109/09637486.2012.676028 22475013

[110] Kawai M Okiyama A Ueda Y. Taste enhancements between various amino acids and IMP. Chem Senses. (2002) 27:739–45. 10.1093/chemse/27.8.739 12379598

[111] Campagnol PCB dos Santos BA Terra NN Pollonio MAR. Lysine, disodium guanylate and disodium inosinate as flavor enhancers in low-sodium fermented sausages. Meat Sci. (2012) 91:334–8. 10.1016/j.meatsci.2012.02.012 22391056

[112] De Klerk A Elings JA Winkel C Tondeur AP Elings J Givaudan S. Use of 4-Substituted Glutamic Acid for Imparting, Enhancing or Modifying Umami and/or Salt Taste in Comestible Product Chosen From Wet Soup and Dehydrated and Culinary Food Patent WO2013060816-A1; EP2770847-A1; US2014272067-A1 (2013).

[113] Amorim M Pereira JO Silva LB Ormenese R Pacheco MTB Pintado M. Use of whey peptide fraction in coated cashew nut as functional ingredient and salt replacer. LWT Food Sci Technol. (2018) 92:204–11. 10.1016/j.lwt.2017.12.075

[114] Jung K. A study on the salty enhancing effect in salad dressing using enzymatically hydrolyzed isolated soy protein. Food Eng Prog. (2019) 23:146–50. 10.13050/foodengprog.2019.23.2.146

[115] Beksan E Schieberle P Robert F Blank I Fay LB Schlichtherle-Cerny H. Synthesis and sensory characterization of novel umami-tasting glutamate glycoconjugates. J Agric Food Chem. (2003) 51:5428–36. 10.1021/jf0344441 12926893

[116] Hong JH Jung DW Kim YS Lee SM Kim KO. Impacts of glutathione maillard reaction products on sensory characteristics and consumer acceptability of beef soup. J Food Sci. (2010) 75:S427–34. 10.1111/j.1750-3841.2010.01783.x 21535516

[117] Halpern BP. Glutamate and the flavor of foods. J Nutr. (2000) 130:910S−4S. 10.1093/jn/130.4.910S 10736351

[118] Jinap S Hajeb P Karim R Norliana S Yibadatihan S Abdul-Kadir R. Reduction of sodium content in spicy soups using monosodium glutamate. Food Nutr Res. (2016) 60:30463. 10.3402/fnr.v60.30463 27356909 PMC4926097

[119] Martínez-Tomé M Murcia MA Mariscal M Lorenzo ML Gómez-Murcia V Bibiloni M. Evaluation of antioxidant activity and nutritional composition of flavoured dehydrated soups packaged in different formats. Reducing the sodium content. J Food Sci Technol. (2015) 52:7850–60. 10.1007/s13197-015-1940-y 26604357 PMC4648876

[120] Baryłko-Pikielna N Kostyra E. Sensory interaction of umami substances with model food matrices and its hedonic effect. Food Qual Prefer. (2007) 18:751–8. 10.1016/j.foodqual.2007.01.002

[121] Manabe M Sakaue R Obata A. Contribution of the retronasal odor of soy sauce to salt reduction. J Food Sci. (2020) 85:2523–9. 10.1111/1750-3841.15332 32654181

[122] Wang SC Tonnis BD Wang ML Zhang SK Adhikari K. Investigation of monosodium glutamate alternatives for content of umami substances and their enhancement effects in chicken soup compared to monosodium glutamate. J Food Sci. (2019) 84:3275–83. 10.1111/1750-3841.14834 31602667

[123] Hsueh CY Tsai ML Liu T. Enhancing saltiness perception using chitin nanofibers when curing tilapia fillets. LWT Food Sci Technol. (2017) 86:93–8. 10.1016/j.lwt.2017.07.057

[124] Danguin TT Salles C Guichard E. Aroma compounds to rescue the taste of healthy foods and beverages. Abstracts of Papers of the American Chemical Society (2016). p. 252.

[125] Lawrence G Salles C Septier C Busch J Thomas-Danguin T. Odour-taste interactions: a way to enhance saltiness in low-salt content solutions. Food Qual Prefer. (2009) 20:241–8. 10.1016/j.foodqual.2008.10.004

[126] Seo H-S Iannilli E Hummel C Okazaki Y Buschhüter D Gerber J. A salty-congruent odor enhances saltiness: functional magnetic resonance imaging study. Hum Brain Mapp. (2013) 34:62–76. 10.1002/hbm.21414 22020878 PMC6870243

[127] Chokumnoyporn N Sriwattana S Prinyawiwatkul W. Saltiness enhancement of oil roasted peanuts induced by foam-mat salt and soy sauce odour. Int J Food Sci Technol. (2016) 51:978–85. 10.1111/ijfs.13048

[128] Chokumnoyporn N Sriwattana S Phimolsiripol Y Torrico DD Prinyawiwatkul W. Soy sauce odour induces and enhances saltiness perception. Int J Food Sci Technol. (2015) 50:2215–21. 10.1111/ijfs.12885

[129] Barnett SM. Sensory Properties, Consumer Perception, and Analytical Assessment of Reformulated Reduced Sodium Ready to Eat Products. (Ph.D.), Washington State University, Ann Arbor, MI, United States (2019).

[130] Barnett SM Sablani SS Tang JM Ross CF. Utilizing herbs and microwave-assisted thermal sterilization to enhance saltiness perception in a chicken pasta meal. J Food Sci. (2019) 84:2313–24. 10.1111/1750-3841.14736 31313314

[131] Kurobayashi Y Fujita A Kubota K. Effects of characteristic volatiles of boiled celery on chicken broth flavor. In: Food Flavour: American Chemical Society (2008). p. 78–86. 10.1021/bk-2008-0988.ch007

[132] Kurobayashi Y Katsumi Y Fujita A Morimitsu Y Kubota K. Flavor enhancement of chicken broth from boiled celery constituents. J Agric Food Chem. (2008) 56:512–6. 10.1021/jf072242p 18163555

[133] Nasri N Beno N Septier C Salles C Thomas-Danguin T. Cross-modal interactions between taste and smell: odour-induced saltiness enhancement depends on salt level. Food Qual Prefer. (2011) 22:678–82. 10.1016/j.foodqual.2011.05.001

[134] Li Y Wan Z Yang X. Salt reduction in liquid/semi-solid foods based on the mucopenetration ability of gum arabic. Food Funct. (2019) 10:4090–101. 10.1039/c8fo02593b 31232415

[135] Kim H Mellema M De Oliveira MC Wolf KM Unilever N Unilever P. Savory Concentrate for Preparing a Savory Product, e.g., Broth, Bouillon, Soup, Sauce or Gravy, Comprises Inorganic Salt, Fat, Psyllium Seed Husk Gum, Glutamate Component, Starch Component, Sugar, Vegetable Matter and Water Patent WO2018137921-A1; AR110845-A1 (2018).

[136] Rama R Chiu N Carvalho Da Silva M Hewson L Hort J Fisk ID. Impact of salt crystal size on in-mouth delivery of sodium and saltiness perception from snack foods. J Texture Stud. (2013) 44:338–45. 10.1111/jtxs.12017

[137] Noort MWJ Bult JHF Stieger M Hamer RJ. Saltiness enhancement in bread by inhomogeneous spatial distribution of sodium chloride. J Cereal Sci. (2010) 52:378–86. 10.1016/j.jcs.2010.06.018

[138] Kuo W-Y Lee Y. Effect of food matrix on saltiness perception-implications for sodium reduction. Compr Rev Food Sci Food Saf. (2014) 13:906–23. 10.1111/1541-4337.12094

[139] Carter BE Monsivais P Drewnowski A. The sensory optimum of chicken broths supplemented with calcium di-glutamate: a possibility for reducing sodium while maintaining taste. Food Qual Prefer. (2011) 22:699–703. 10.1016/j.foodqual.2011.05.003

[140] Kim H Lee J Kim B. Development of an initial lexicon for and impact of forms (cube, liquid, powder) on chicken stock and comparison to consumer acceptance. J Sens Stud. (2017) 32:e12251. 10.1111/joss.12251

[141] Vosti SA Kagin J Engle-Stone R Luo H Tarini A Clermont A. Strategies to achieve adequate vitamin A intake for young children: options for Cameroon. Ann N Y Acad Sci. (2020) 1465:161–80. 10.1111/nyas.14275 31797386 PMC7187426

[142] Dold S Zimmermann MB Jeroense F Zeder C Habeych E Galaffu N. Iron bioavailability from bouillon fortified with a novel ferric phytate compound: a stable iron isotope study in healthy women (part II). Sci Rep. (2020) 10:5339. 10.1038/s41598-020-62307-1 32210349 PMC7093532

